# Insights into the Regulation of Tumor Angiogenesis by Micro-RNAs

**DOI:** 10.3390/jcm8122030

**Published:** 2019-11-20

**Authors:** Patrizia Leone, Alessio Buonavoglia, Rossella Fasano, Antonio Giovanni Solimando, Valli De Re, Sebastiano Cicco, Angelo Vacca, Vito Racanelli

**Affiliations:** 1 Department of Biomedical Sciences and Human Oncology, University of Bari Medical School, 70124 Bari, Italy; alessio.buonavoglia85@gmail.com (A.B.); rossella.fasano.93@gmail.com (R.F.); antoniogiovannisolimando@gmail.com (A.G.S.); sebacicco@gmail.com (S.C.); angelo.vacca@uniba.it (A.V.); vito.racanelli1@uniba.it (V.R.); 2 Medical Oncology Unit, IRCCS Istituto Tumori “Giovanni Paolo II” of Bari, Viale Orazio Flacco, 65, 70124 Bari, Italy; 3 Bio-Proteomics Facility, Department of Translational Research, Centro di Riferimento Oncologico di Aviano (CRO) IRCCS, 33081 Aviano (PN), Italy; vdere@cro.it

**Keywords:** tumor angiogenesis, microRNAs, endothelial cells, anti-angiogenic therapies

## Abstract

One of the hallmarks of cancer is angiogenesis, a series of events leading to the formation of the abnormal vascular network required for tumor growth, development, progression, and metastasis. MicroRNAs (miRNAs) are short, single-stranded, non-coding RNAs whose functions include modulation of the expression of pro- and anti-angiogenic factors and regulation of the function of vascular endothelial cells. Vascular-associated microRNAs can be either pro- or anti-angiogenic. In cancer, miRNA expression levels are deregulated and typically vary during tumor progression. Experimental data indicate that the tumor phenotype can be modified by targeting miRNA expression. Based on these observations, miRNAs may be promising targets for the development of novel anti-angiogenic therapies. This review discusses the role of various miRNAs and their targets in tumor angiogenesis, describes the strategies and challenges of miRNA-based anti-angiogenic therapies and explores the potential use of miRNAs as biomarkers for anti-angiogenic therapy response.

## 1. Introduction

During embryogenesis, the vascular network develops through vasculogenesis (de novo production of endothelial cells and their assembly and further differentiation into new blood vessels) and angiogenesis (sprouting of new blood vessels from pre-existing ones). Thereafter, the vasculature becomes largely quiescent. In the adult, angiogenesis can be transiently activated, as occurs during the reproductive cycle in females. However, angiogenesis is derailed in various diseases, especially cancer [1]. A pre-requisite of tumor development is the rapid formation of a vascular network to sustain the high proliferative rate of cancer cells. This is achieved via the high-level secretion by tumor and stromal cells of pro-angiogenic factors that create an angio-competent milieu [2]. Supported by angiogenesis, tumors are able to obtain the nutrients and oxygen required for their growth. The newly formed vascular network also facilitates the removal of metabolic waste and carbon dioxide from the tumor microenvironment while providing a route for tumor dissemination/metastasis [3], promoting metabolic deregulation [4], and enhancing cancer stem cell persistence [5]. Thus, given the essential role of angiogenesis in tumor growth, the development of new strategies for cancer treatment requires a detailed understanding of its regulation.

Among the regulators of tumor angiogenesis are microRNAs (miRNAs), which can act as anti-angiogenic but also as pro-angiogenic factors [6,7]. miRNAs are small (21–25 nucleotides) non-coding RNAs that negatively regulate gene expression [8]. TargetScanS analyses indicate that one third of human genes is subject to miRNA control [9] highlighting the role of miRNAs as critical regulators of various processes including key steps of cancer, such as tumor growth, metastasis, angiogenesis, and drug resistance [6,7,10,11,12]. In particular, miRNAs are so involved in the regulation of angiogenesis that global miRNA depletion suppresses the angiogenic process [13]. miRNAs regulate angiogenesis directly, by influencing the activity of endothelial cells, or indirectly, by modulating the expression of proteins that promote or inhibit vessel growth [7]. Consequently, miRNAs have generated interest as promising targets in novel anti-angiogenic therapies.

This review discusses the role of miRNAs and their cellular targets in tumor angiogenesis, describes the strategies and challenges of miRNA-based anti-angiogenic therapies, and explores the potential use of miRNAs as biomarkers for anti-angiogenic therapy response.

## 2. Tumor Angiogenesis

Angiogenesis is a complex, multi-step process that involves the activation, migration, proliferation, and differentiation of endothelial cells followed by their reorganization into new tubular structures [14,15,16]. Every step is controlled by numerous pro- and anti-angiogenic factors.

Pro-angiogenic factors include vascular endothelial growth factors (VEGFs), fibroblast growth factor (FGF)-1 and -2, and platelet-derived endothelial cell growth factor (PDGF), all of which induce endothelial cell proliferation and migration, as well as angiopoietins, which cooperate with other angiogenic factors to control the activation status of endothelial cells, as well as endothelial tube formation, by binding to Tie-2 tyrosine kinase receptors. VEGF-A, -B, -C, and -D, the most important regulators of angiogenesis [17], bind to three VEGF tyrosine kinase receptors, VEGFR-1, VEGFR-2, and VEGFR-3. These receptors are specific for endothelial cells and their expression is strongly influenced by the Notch signaling pathway [18,19]. VEGF-A is the prototypic member of the VEGF superfamily, and along with its receptors, VEGFR-2 and VEGFR-1, forms the best-characterized signaling pathway involved in angiogenesis [20].

Anti-angiogenic factors include components and proteolytic fragments of the extracellular matrix, such as the extracellular matrix glycoprotein thrombospondin-1 (TSP1) [21], the endostatin, a cleavage product of collagen XVIII [22], and canstatin and tumstatin, two proteolytic fragments of collagen IV [23,24]. Other important endogenous inhibitors of angiogenesis are soluble factors such as interferon-α and -β, and angiostatin, a cleavage product of plasmin [25,26].

The differential expression, release, and activation of the various pro- and anti-angiogenic factors but especially the balance between them, finely regulates angiogenesis under physiological and pathological conditions. Under physiological conditions, stromal cells, endothelial cells, and secreted molecules act in concert within a dynamic system which constantly changes with a tendency towards anti-angiogenic factors, thus maintaining the quiescence of the vasculature. Within tumors, the balance is tilted in favor of pro-angiogenic factors, the “angiogenic switch” is turned on and the formation of new blood vessels accordingly induced. Pro-angiogenic factors can also be secreted by tumor cells themselves and by tumor-infiltrating inflammatory/immune cells [27]. The “angiogenic switch” is an essential step in tumor development that can occur at different stages in tumorigenesis, depending on the nature of the tumor and its microenvironment [1]. Mechanical and metabolic stress, and genetic mutations (for instance, expression of oncogenes or deletion of tumor-suppressor genes that regulate the production of angiogenesis regulators) can also influence the balance [28]. As noted above, the tumor vasculature serves to provide the tumor with an adequate supply of oxygen, nutrients, and an effective system of metabolic waste removal. Moreover, in combination with lymphangiogenesis, neovascularization provides an “escape route” for tumor cells into the bloodstream, thus contributing to tumor dissemination and metastasis [29].

Tumor angiogenesis differs significantly from physiological angiogenesis. First, tumor angiogenesis is persistent and is characterized by the continuous growth of new tumor-supporting blood vessels. Moreover, the tumor vasculature is itself characterized by unique features that distinguish it from the normal vasculature. Thus, tumor blood vessels are dilated, irregularly shaped, and leaky and form a disorganized, chaotic vascular network that is not composed of the distinct venules, arterioles, and capillaries that make up the normal vascular system. Endothelial cells of tumor vessels exhibit structural and functional alterations as well as abnormal pericyte interactions. In the normal vasculature, pericytes surround the endothelium and form gap junction connections with endothelial cells, whereas those associated with the tumor loosely interact with endothelial cells and are often absent [30]. Tumor blood vessels mature slowly and their high permeability explains the irregular and oscillatory flow of blood to the tumor. Seaman et al. compared the gene expression patterns of endothelial cells derived from the blood vessels of eight normal resting tissues, five tumors, and regenerating liver. They identified 13 genes whose expression was at least 10-fold higher in tumor than in normal endothelium and that were not expressed in the angiogenic endothelium of regenerating liver [31].

A further aspect of tumor angiogenesis is the ability of tumor cells to undergo vasculogenic mimicry, in which they dedifferentiate to acquire endothelial-like properties [32]. Tumor cells can form matrix-rich vessel-like networks composed of channels that are not true vessels and do not arise from preexisting vessels. Studies of tumor-tissue sections showed tumor cell clusters separated by channel-like spaces that mimic the function of blood vessels, serving as intratumoral fluid conductors and containing erythrocytes and plasma [33]. Moreover, both endothelial cells and tumor cells can line the luminal surface of tumor-associated blood vessels, resulting in so-called mosaic vessels [34]. Since these tumor cells are directly exposed to blood flow, their detachment results in their direct entry into the bloodstream. A recent clinical meta-analysis showed that patients whose tumors show signs of vasculogenic mimicry have a poor clinical outcome [35].

Vasculogenic mimicry can be induced by hypoxia, a condition that ensues when the tumor mass expands such that tumor cells are far removed from blood vessels and are therefore deprived of adequate oxygen and nutrient supplies. The hypoxic microenvironment cooperates with oncogenic processes to trigger angiogenesis [36]. The most important transcriptional factor driving hypoxia-induced events is hypoxia-inducible factor-1 (HIF-1). HIF-1 consists of α and β subunits. HIF-1α is an oxygen-sensing factor that stimulates angiogenesis by positively regulating the expression of pro-angiogenic mediators, including VEGF-A. In normoxia, low levels of HIF-1α are ensured by the ubiquitin proteasome-dependent degradation mediated by the von Hippel–Lindau (VHL) protein, a component of the E3 ubiquitin ligase complex. Under hypoxic condition, HIF-1α degradation is inhibited, resulting in an increase of HIF-1α levels and the promotion of angiogenesis [37].

## 3. MicroRNAs

**Figure 1:**
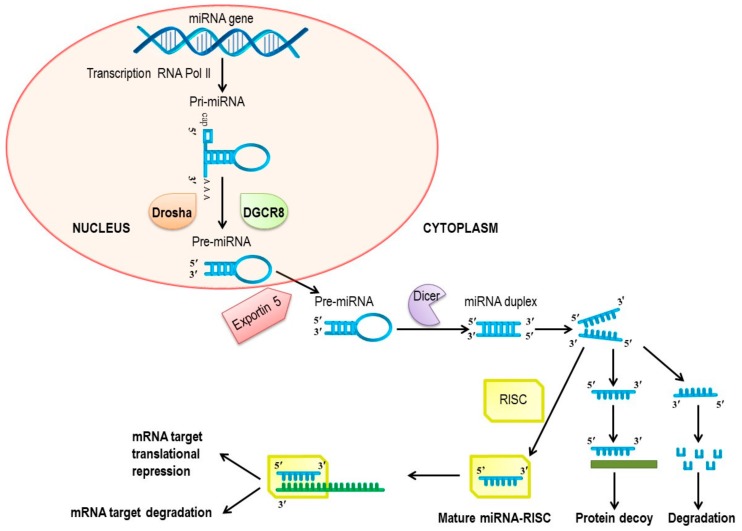
Biogenesis of miRNAs. miRNAs: microRNAs; mRNA: messenger RiboNucleic Acid; pri-miRNAs: primary miRNA transcripts; Drosha: Drosha ribonuclease III; Dicer: Dicer 1 ribonuclease III; DGCR8: DiGeorge syndrome critical region 8; pre-miRNA: precursor miRNA; RISC: RNA-induced silencing complex.

MicroRNAs (miRNAs) are small, single-stranded, endogenous non-coding RNAs with transcriptional and post-transcriptional gene silencing functions. MiRNAs typically bind to the 3′-untranslated region (3′-UTR) of their target messenger RiboNucleic Acid (mRNA) or, less frequently, to the 5′-UTR and coding region [38]. The biogenesis of miRNAs starts in the nucleus, where miRNA genes are transcribed into primary miRNA transcripts (pri-miRNAs) by RNA polymerase II (Pol II) [39]. Pri-miRNAs are characterized by one or more specific long-hairpin structures that include a 3′-polyadenylation tail and a 5′-cap structure. The further two-step processing of pri-mRNAs is regulated by the RNase III enzymes Drosha and Dicer [6]. In the nucleus, the interaction of Drosha with the RNA-binding protein DGCR8 (DiGeorge syndrome critical region 8) results in a complex that recognizes and cleaves the pri-miRNA into a 60–70 nucleotide (nt) long stem loop precursor miRNA (pre-miRNA) that is subsequently exported to the cytoplasm by an exportin-5-dependent mechanism [40]. The cytoplasmic pre-miRNA is further processed by Dicer, which cleaves the stem-loop to generate a double-stranded, 18–25 nt long miRNA [41]. Unwinding of the double-stranded miRNA is followed by the incorporation of the single-strand guide (mature) miRNA into the RNA-induced silencing complex (RISC), while the remaining strand is degraded. In the RISC, the 5′-region of the guide miRNA binds to the 3′-UTR of the target mRNA to regulate gene expression by inducing mRNA degradation or translational repression. Mature miRNA can also bind directly to RNA-binding proteins in a sequence-dependent manner, thereby preventing the attachment of these proteins to their RNA targets. This decoy activity of miRNA is RISC-independent (Figure 1) [42].

## 4. Tumor Angiogenesis and miRNAs

miRNAs have a wide range of cellular functions. Of interest to this review is their ability to modulate the expression of pro- and anti-angiogenic factors and to regulate vascular endothelial cell function [6,7]. Vascular-associated microRNAs (angiomiRs) can be either pro- (Table 1) or anti-angiogenic (Table 2) [43]. The different miRNAs in each of these groups are discussed below.

## 5. Pro-Angiogenic miRNAs

### 5.1. miR-155

miR-155 downregulates the von Hippel–Lindau syndrome (VHL) gene, implicated in the proteasome-mediated degradation of HIF-1α under normal oxygen levels. Under hypoxic conditions or following the loss of VHL function, HIF-1α is stabilized, resulting in the induction of angiogenesis [37,114,115]. The upregulation of miR-155 is associated with lymph node metastasis, angiogenesis induction, and a poor prognosis of patients with triple negative breast cancer (TNBC) [44,45] or renal cell carcinoma [46]. High levels of miR-155 also increase human umbilical vein endothelial cell (HUVEC) proliferation, invasion, migration, and tube formation [44,116]. A recent study demonstrated that metastatic melanoma cell lines release and use exosomes to transfer miR-155 in fibroblasts, thus inducing the pro-angiogenic switch. These exosomes enhance the expression levels of VEGF-A, FGF-2, and matrix metalloproteinase-9 (MMP-9) [47].

### 5.2. miR-566

The overexpression of miR-566 in human glioma cell lines activates the epidermal growth factor receptor (EGFR) pathway, through direct targeting of VHL. miR-566 upregulation is also associated with a reduction of VHL expression, high VEGF levels, the high-level expression of HIF-1α, and an increased invasion and migration ability of glioblastoma cells [48,49]. Conversely, miR-566 inhibition slows the proliferation of glioma cells and sensitizes them to anti-EGFR agents [49].

### 5.3. miR-210

The expression of miR-210 is strictly linked to hypoxia [117]. This miRNA regulates hypoxia-induced intracellular pathways, including those leading to angiogenesis in breast and ovarian cancers as well as in many other tumors [50,51]. In response to ischemic injury in the brain, miR-210 activates angiogenesis by triggering the Notch pathway. It also induces endothelial cells to migrate and form capillary-like structures on Matrigel [52]. A further study showed that miR-210 modulates the endothelial cell response to hypoxia and promotes angiogenesis by targeting the receptor tyrosine kinase ligand ephrin-A3 (EFNA3), an anti-angiogenesis factor [53].

In hepatitis B virus (HBV)-related hepatocellular carcinoma (HCC), miR-210 expression was shown to progressively increase from normal liver to tumor formation and to change depending on the stage of liver disease. High-level expression of miR-210 is associated with a more aggressive tumor phenotype and a poor prognosis. Both in vitro and in vivo studies have shown that miR-210 promotes HCC angiogenesis by negatively regulating the expression of FGF receptor-like 1, a decoy FGF receptor and inhibitor of angiogenesis in HCC [54].

### 5.4. miR-21

miR-21 targets phosphatase and tensin homolog deleted on chromosome ten (PTEN) and induces angiogenesis through the upregulation of HIF-1α and VEGF expression via the activation of AKT (RAC-Alpha Serine/Threonine-Protein Kinase) and extracellular regulated kinase (ERK) pathways, as demonstrated in prostate cancer cell lines [55], glioma [56], pancreatic cancer cell lines [57], breast cancer [58], and HCC [59]. Another target of miR-21 is the tumor suppressor programmed cell death gene 4 (Pdcd4). High levels of miR-21 are associated with the downregulation of Pdcd4 and the stimulation of invasion, intravasation, and metastasis in colorectal cancer [60].

### 5.5. miR-182

Under hypoxic conditions, miR-182 stimulates angiogenesis by increasing VEGF and HIF-1α expression. In breast cancer cells, it targets the E3 ubiquitin-protein ligase FBXW7 (F-box and WD repeat domain-containing 7) [61], whereas in prostate cancer cells its targets are two negative regulators of HIF-1 signaling: PHDs (prolyl hydroxylase domain enzymes) and FIH1 (factor inhibiting HIF-1) [62].

### 5.6. miR-296

miR-296 contributes significantly to angiogenesis by directly targeting hepatocyte-growth-factor-regulated tyrosine kinase substrate (HGS) mRNA, causing a decrease in HGS levels and thereby a reduction in the HGS-mediated degradation of VEGFR-2 and PDGF receptor beta [63]. In primary tumor endothelial cells isolated from human brain tumors (gliomas), miR-296 levels are higher than in normal brain endothelial cells [63].

### 5.7. miR-17-92 Cluster

The miR-17-92 locus encodes a cluster of seven microRNAs: miR-17-5p, miR-17-3p, miR-18a, miR-19a, miR-20a, miR-19b-1, and miR-92-1 [118], which are potent promoters of tumor angiogenesis. Expression of the miRNAs in this cluster is directly activated by the transcription factor c-Myc [119,120]. In endothelial cells, VEGF stimulates cluster expression through the ERK/ELK1 (ETS Transcription Factor ELK1) activation pathway. Upregulation of the miR-17-92 cluster is associated with the downregulation of anti-angiogenic thrombospondin-1 (Tsp1) and related proteins, such as connective tissue growth factor [64]. Another target of the miR-17-92 cluster involved in angiogenesis regulation is PTEN [64]. In their study of lung cancer cell lines, Taguchi et al. identified HIF-1α as target of the miR-17-92 cluster and suggested the existence of a feedback loop between c-Myc and HIF-1α via these miRNAs [65].

### 5.8. Lethal (Let)-7b and -7f

Among the members of the let-7 family, let-7b and let-7f promote angiogenesis by suppressing two important inhibitors of cellular migration: Tissue inhibitor of metalloproteinase 1 (TIMP-1) and Tsp 1 and 2 [66,67]. In prostate cancer, let-7b regulates macrophage polarization and enhances tumor-associated macrophages to stimulate angiogenesis by modulating the expression profile of inflammatory cytokines such as interleukin (IL)-12, IL-23, IL-10, and tumor necrosis factor-α [68].

### 5.9. miR-378

Higher-level expression of miR-378 in ovarian cancer cells than in normal ovarian surface epithelial cells has been described, together with an associated dysregulation of several angiogenesis genes, such as activated leukocyte cell adhesion molecule (ALCAM), EH-domain containing 1 (EHD1), serine/threonine-protein kinase tousled-like 1 (TLK1), and the transcriptional repressor ELK3 (ETS Transcription Factor ELK3) [69]. In vitro and in vivo studies have shown that miR-378 regulates endothelial cell function and amplifies VEGF activity to promote angiogenesis by repressing the tumor suppressors Sufu (suppressor of fused) and Fus-1 (nuclear fusion protein 1), as shown in a glioblastoma cell line [70], in non-small cell lung cancer (NSCLC) patients with brain metastases [71], and in bladder cancer [72].

### 5.10. miR-221 and miR-222

miR-221/222 can positively or negatively modulate angiogenesis [121]. In human glioblastoma and glioma, these miRNAs promote angiogenesis [73,74]. In glioblastoma cells, miR-221/222 are expressed at high levels and their downregulation results in a reduction of tumor invasion, migration, proliferation, and angiogenesis in association with decreased levels of MMP-2, MMP-9 and VEGF, and inactivation of the Janus kinase (JAK)/signal transducer of activation (STAT) pathway by the upregulation of SOCS3 (suppressor of cytokine signaling-3) [73]. Yang et al. demonstrated that miR-221/222 are significantly upregulated in glioma cells and promote angiogenesis by targeting TIMP2, an anti-angiogenic factor that inhibits tube formation by halting the activity of MMPs [74].

## 6. Anti-Angiogenic miRNAs

### 6.1. miR-221 and miR-222

miR-221 and miR-222 are highly expressed in endothelial cells, where they inhibit c-Kit expression and, consequently, the angiogenic activity of its ligand, stem cell factor (SCF), resulting in impaired endothelial cell migration, proliferation, and the ability to form new capillaries [75]. Both c-Kit and VEGFR2 are targets of miR-221/-222 and of the small-molecule anti-tumor agent sunitinib. Khella et al. showed that the upregulation of miR-221/-222 is associated with a decrease in VEGFR-2 expression and the poor survival of patients with metastatic renal cell carcinoma treated with sunitinib [76]. miR-221/-222 also target the transcription factor E26 transformation-specific sequence-1, thus decreasing angiotensin II-induced HUVEC migration [77].

miR-221 is involved in vascular remodeling and is essential for endothelial tip cell behavior during vascular development. In an embryonic zebrafish model, miR-221 was shown to inhibit sprouting endothelial cells through the repression of two distinct target transcripts: Cyclin-dependent kinase inhibitor 1b and the p85-alpha regulatory subunit of phosphoinositide-3-kinase [78]. It also influences the angiogenic phenotype in endothelial cells by downregulating ZEB2 (Zinc finger E-box-binding homeobox 2), a transcriptional repressor of the growth-arrest-specific homeobox (GAX) [79]. In both in vitro and in vivo models, ZEB2 downregulation by miR-221 is associated with the upregulation of GAX as well as the inhibition of endothelial cell proliferation [80,81] and angiogenesis [81]. Kontomanolis et al. described high plasma levels of miR-221 in patients with breast cancer associated with poor vascular maturation and a high risk to develop metastasis [82].

In inflammation, miR-222 mediates neovessel formation by inhibiting the expression of STAT5A (signal transducer and activator of transcription 5A) [83].

### 6.2. miR-126

miR-126, along with miR-221/-222, is highly expressed in endothelial cells [67]. During embryogenesis it has pro-angiogenic action, including endothelial cell proliferation, migration, survival, and the regulation of blood vessel integrity [122]. In response to VEGF and basic fibroblast growth factor (bFGF), miR-126 promotes angiogenesis by directly suppressing negative regulators of the VEGF pathway, including sprouty-related EVH1 domain containing protein 1 and phosphoinositide-3-kinase regulatory subunit 2 (PIK3R2) [122]. However, during tumor angiogenesis, miR-126 acts as anti-angiogenic factor [123]; as such, it is downregulated in many tumors, including esophageal [84], breast [85], and cervical [86] cancers. Specifically, in esophageal cancer and esophageal cancer cell lines miR-126 expression is significantly lower than in healthy tissues, while the expression of its target, VEGF-A, is high [84]. In breast cancer, VEGF-A and PIK3R2 are targets of miR-126 and are downregulated in tumors in which the VEGF/PI3K (phosphoinositide 3-kinase)/AKT signaling pathway is activated [85]. In cervical cancer, miR-126 inhibits angiogenesis, microvessel density, and tumor growth by targeting expression of the pro-angiogenic gene adrenomedullin (ADM) [86].

### 6.3. Let-7a

Let-7a inhibits tube formation and reduces the migration rate of HUVECs by directly targeting transforming growth factor beta (TGFβ) receptor III, resulting in a defective TGFβ signaling pathway in these cells [87]. Sureban et al. demonstrated that, in pancreatic cancer and HCC, doublecortin-like kinase 1 (DCLK1), a putative marker of intestinal and pancreatic stem cells, regulates the pluripotency and expression of angiogenic factors via miRNA-dependent mechanisms. The downregulation of DCLK1 upregulates several tumor suppressor miRNAs, including let-7a, thus inhibiting tumor growth, metastasis, and angiogenesis [88,89,90].

### 6.4. miR-328

miR-328 targets CD44 (cluster of differentiation 44), a transmembrane glycoprotein involved in numerous cellular functions, including cell–cell and cell–matrix interactions, lymphocyte activation and homing, hematopoiesis, tumor metastasis, and cell migration [124,125,126]. The overexpression of miR-328 suppresses CD44 expression and reduces endothelial cell activity, tubulogenesis, and blood vessel formation in MT1 breast cancer cells [91].

### 6.5. miR-135a

Tumor suppressive miR-135a is downregulated in several cancers. In gastric cancer, its expression inhibits tumor growth, migration, invasion, and angiogenesis by targeting the focal adhesion kinase pathway, which regulates VEGF signaling [92]. In NSCLC, miR-135a suppresses cell proliferation, migration, invasion, apoptosis, and angiogenesis by blocking the insulin-like growth factor (IGF)-1/PI3K/AKT signaling pathway. High levels of miR-135a are associated with the low-level expression of IGF-1, PI3K, AKT, IL-8, VEGF, and bFGF [93].

### 6.6. miR-29b

The levels of miR-29b are downregulated in various cancers, including endometrial carcinoma, breast cancer, pancreatic ductal adenocarcinoma, and hepatocellular carcinoma [94,95,96,97]. Systemic administration of miR-29b in mouse xenograft tumor models suppresses tumor vascularization, as well as tumor cell proliferation, invasion, and m igration [94,95,96]. In endometrial carcinoma, miR-29b expression simultaneously represses angiogenesis and tumorigenesis, both in vitro and in vivo, by negatively modulating VEGF expression and the mitogen-activated protein kinase (MAPK)/ERK and PI3K/AKT signaling pathways [94]. Compared with adjacent normal tissues, miR-29b expression is downregulated in breast cancer and endometrial carcinoma tissues, whereas VEGFA, ERK, and AKT3 are upregulated [94,96]. miR-29b was also shown to negatively regulate VEGF-A expression in pancreatic ductal adenocarcinoma [97]. In hepatocellular carcinoma, miR-29b directly suppresses MMP-2 expression and, in turn, impairs VEGFR-2-signaling in endothelial cells. Its overexpression inhibit angiogenesis and tumorigenesis in vivo and represses the ability of hepatocellular carcinoma cells to promote capillary tube formation of endothelial cells [95].

### 6.7. miR-206

miR-206 inhibits angiogenesis by downregulating VEGF. A negative correlation is found between miR-206 and VEGF levels in laryngeal squamous cell carcinoma [98], TNBC [99], and NSCLC [100]. In TNBC, the main targets of miR-206 are VEGF, MAPK3, and the transcription factor SOX9 (Sry-type HMG box9) [99]. In NSCLC, which is characterized by intense angiogenesis, miR-206 blocks 14-3-3ζ/STAT3/HIF-1α/VEGF signaling [100]. By targeting c-Met/PI3k/AKT/mTOR (mammalian target of rapamycin) signaling, miR-206 inhibits the hepatocyte-growth-factor-induced epithelial-mesenchymal transition, the migration and invasion of lung cancer cells, as well as HUVEC migration and capillary tube formation [101,127].

### 6.8. miR-140-5p

miR-140-5p plays a tumor suppressive role in colorectal cancer and glioma. It directly targets VEGF-A, inhibiting tumor progression and angiogenesis by regulating the VEGF-A/MMP-2 signaling [102,103]. In glioblastoma, VEGF is located upstream of MMP-2 in the ERK/MAPK pathway such that VEGF affects MMP-2 expression and therefore tumor cell proliferation and neovascularization [104]. In advanced colorectal cancer, miR-140-5p levels are significantly lower than in normal tissues and their downregulation correlates with higher-stage disease and poorer overall survival [103].

### 6.9. miR-497

In colorectal cancer, miR-497 targets VEGF-A, leading to blockade of the VEGF-A/ERK/MMP-9 signaling pathway and the attenuation of tumor angiogenesis, invasion, and metastasis [105]. Similarly, in breast cancer cells, miR-497 downregulates VEGF and HIF-1α levels, thus reducing tumor growth and angiogenesis, as demonstrated both in vitro and in vivo. In HUVECs treated with conditioned medium from breast cancer cells transfected with a miR-497 mimic, both tube formation and branch point formation are decreased [106]. In renal cell carcinoma, in vitro experiments have shown that miR-497 suppresses VEGFR2 expression and negatively regulates the MEK/ERK and p38 MAPK pathways [107].

### 6.10. miR-377

In tumor tissue and in the serum of patients with esophageal squamous cell carcinoma, miR-377 expression is significantly downregulated. Its expression reduces tumor growth and angiogenesis by targeting VEGF and CD133, a marker of tumor-initiating cells. Negative correlations between miR-377 levels and tumor stage, distant metastasis, and chemoradiotherapy resistance have been reported [108]. In glioblastoma cells, miR-377 targets specific protein 1 (Sp1), resulting in the inhibition of tumor cell proliferation and invasion [109]. A link to angiogenesis is suggested by the fact that Sp1 overexpression is associated with the upregulation of VEGF in human pancreatic cancer [128], gastric cancer [129], and in human fibrosarcoma cell lines [130].

### 6.11. miR-218

miR-218 reduces VEGF expression in prostate cancer by acting on the mTOR component RICTOR (rapamycin-insensitive companion of mammalian target of rapamycin) and by blocking the RICTOR/mTOR/HIF-1/VEGF signaling pathway [110]. In gastric cancer, two targets of miR-218 have been identified: Angiopoietin-2 and ROBO1 (roundabout guidance receptor 1); their downregulation results in a reduction of tumor proliferation, invasion, and angiogenesis [111,112]. Moreover, low levels of miR-218 result in endothelial cell sprouting, motility, and tube formation [112].

### 6.12. miR-134

miR-134 binds to the 3′-UTR of the VEGF-A and VEGFR-1 genes. Its expression is strongly downregulated in human osteosarcoma cell lines and tissues whereas its overexpression inhibits the proliferation and angiogenesis of osteosarcoma cells by targeting VEGF-A/VEGFR-1-AKT signaling [113].

## 7. Clinical Implications

As angiogenesis plays a critical role in tumor growth and progression, a number of anti-angiogenic agents have been developed, but the response rates have been low. Among the disadvantages of anti-angiogenesis therapy are the lack of predictive biomarkers to distinguish responders from non-responders, drug resistance, and the involvement of many factors in the regulation of angiogenesis [131,132]. However, the high stability and accessibility of miRNAs in biofluids, their altered expression in tumor angiogenesis, and their capacity to target multiple transcripts [42] suggest that miRNAs can serve as predictive biomarkers while their inhibition of angiogenesis may provide a therapeutic option in many different cancers. miRNA-based anti-angiogenic treatments may also be used in combination with immunotherapies, such as immune-checkpoint inhibitors, vaccines, and adoptive cell transfers, to boost adaptive immune response [133]. This synergistic combination is more likely to succeed in patients who either develop resistance to single approaches or do not respond at all [134,135,136].

### 7.1. miRNAs as Biomarkers of the Anti-Angiogenic Therapy Response

High levels of circulating miR-126 are associated with a worse response to therapy in patients with metastatic colorectal cancer treated with first-line chemotherapy combined with bevacizumab, an anti-VEGF antibody [137]. The overexpression of miR-378 may predict the risk of developing brain metastasis in NSCLC patients [71] and is associated with poor progression-free survival in patients with recurrent ovarian cancer treated with bevacizumab [69]. In patients with metastatic renal cell carcinoma, decreased tissue levels of miR-155 are significantly linked to an increased time to progression and resistance to the tyrosine kinase inhibitor sunitinib [138].

### 7.2. miRNAs as Therapeutics Against Tumor Angiogenesis

The use of miRNAs in the therapeutic targeting of tumor angiogenesis has been explored by the delivery of antagomiRs (antisense oligonucleotides, locked nucleic acid antimiR constructs, miRNA sponges, miR-Masks) or miRNA mimics (substitutes for the loss of expression of a tumor-suppressor miRNA) into tumor cells or endothelial cells [42]. The goal is to limit pathological neovascularization by suppressing pro-angiogenic miRNAs or increasing anti-angiogenic miRNAs, respectively.

As naked miRNAs are hydrophilic, their passage through the cell membranes and protection from degradation by serum nucleases is achieved by the use of delivery systems, including chemically modified viral particles or oligonucleotides, liposomes, polymers, hydrogels and nanoparticles, all of which have been tested in tumor-bearing mice models [7,42]. Cyclic arginine-glycine-aspartic acid peptide (cRGD)-coupled nanoparticles have been widely used to efficiently target α_v_β_3_ integrins on the surface of endothelial cells. Administration of anti-miR-132, anti-miR-296, or miR-7 mimics using this system was shown to strongly block angiogenesis and significantly reduce the tumor burden in xenograft mouse models [139,140,141].

The intracellular delivery of anti-angiogenic miRNAs with low toxicity has been achieved using liposomes modified with acetylated polyethylenimine (PEI, polycation liposomes). The injection of miR-125b within PEI liposomes into a tumor xenograft mouse model resulted in a decrease of VE-cadherin expression by endothelial cells, the formation of non-functional blood vessels, and the inhibition of tumor growth [142].

Cell-derived membrane vesicles, such as exosomes and microvesicles, are a novel, highly promising class of miRNA delivery systems. In contrast to synthetic nanoparticles, these vesicles are endogenous carriers and are thus of very low toxicity and low immunogenicity (particularly if harvested from autologous cells). Moreover, they specifically recognize their target cells with reduced off-target effects [7]. For instance, microvesicles were recently shown to be effective carriers in VEGF-targeted therapy in gastric cancer. Microvesicles derived from miR-29-overexpressing human embryo kidney epithelial 293 cells transfected with synthesized miRNA mimics efficiently transport miR-29 into human gastric cells, thereby suppressing VEGF secretion and decreasing vascular cell growth, metastasis, and tube formation [143].

## 8. Conclusions and Perspectives

Further studies of the regulatory role of miRNAs in tumor angiogenesis and the use of miRNAs as predictive biomarkers for anti-angiogenic therapy or as therapeutics to target tumor angiogenesis may lead to new strategies in cancer treatment. The changes in miRNA expression during the progression of many tumors suggest the use of miRNAs as biomarkers in cancer diagnosis and treatment and as prognostic indicators. Moreover, miRNA-based treatments may be used alone or in combination with current targeted therapies to downregulate angiogenesis and enhance disease response. However, the clinical translation of miRNA-based therapies has thus far been hampered by the toxicity, off-targets effects, biological instability, and poor cellular uptake of miRNAs. A challenge of miRNA-based therapy is tissue-specific delivery, minimizing off-target effects, and optimizing therapeutic doses.

## Figures and Tables

**Table 1:** Summary of pro-angiogenic miRNAs involved in tumor angiogenesis regulation.

miRNAs	Targets	Tumor	Function	References
miR-155	VHL	Triple negative breast cancer, renal cell carcinoma, melanoma cell lines	↑ proliferation, invasion, migration and tube formation ↑ VEGF-A, FGF-2 and MMP9	[44,45,46,47]
miR-566	VHL	Glioblastoma, glioma cell line	↑ EGFR pathway, invasion and migration ↑ VEGF, HIF-1α	[48,49]
miR-210	EFNA3 FGFRL1	Breast cancer, ovarian cancer, HBV-related hepatocellular carcinoma	↑ Notch pathway ↑ endothelial cell migration and capillary-like structure formation	[50,51,52,53,54]
miR-21	PTEN Pdcd4	Prostate cancer cell lines, glioma, pancreatic cancer cell lines, breast cancer, hepatocellular carcinoma, colorectal cancer	↑ AKT and ERK1/2 signaling pathways ↑ VEGF, HIF-1α	[55,56,57,58,59,60]
miR-182	FBXW7 PHD FIH1	Breast cancer, prostate cancer	↑ VEGF, HIF-1α	[61,62]
miR-296	HGS	Glioma	↑ VEGFR-2, PDGFRβ	[63]
miR-17-92	Tsp1 CTGF PTEN HIF-1α	Lung cancer cell lines	Endothelial cell activation	[64,65]
let-7b/-7f	TIMP Tsp1/2	Prostate cancer	↑ endothelial cell migration	[66,67,68]
miR-378	ALCAM EHD1 TLK1 ELK3 Sufu Fus-1	Ovarian cancer, non-small cell lung cancer, glioblastoma cell lines, bladder cancer	endothelial cell function regulation ↑ VEGF activity	[69,70,71,72]
miR-221/222	SOCS3 TIMP2	Glioblastoma, glioma	↑ MMP2, MMP9, VEGF ↑ tube formation ↑ JAK/STAT pathway	[73,74]

Abbreviations: miRNAs: microRNAs; VHL: von Hippel–Lindau; VEGF-A: vascular endothelial growth factor-A; FGF-2: fibroblast growth factor-2; MMP-9: matrix metalloproteinase-9; EGFR: epidermal growth factor receptor; HIF-1α: hypoxia-inducible factor-1 alfa; EFNA3: receptor tyrosine kinase ligand ephrin-A3; FGFRL1: FGF receptor-like 1; HBV: Hepatitis B virus; PTEN: tensin homolog deleted on chromosome ten; Pdcd4: programmed cell death gene 4; ERK: extracellular regulated kinase; FBXW7: F-box and WD repeat domain-containing 7; PHD: prolyl hydroxylase domain enzyme; FIH1: factor inhibiting HIF-1; HGS: hepatocyte-growth-factor-regulated tyrosine kinase substrate; VEGFR-2: vascular endothelial growth factor receptor2; PDGFRβ: platelet-derived endothelial cell growth factor receptor beta; Tsp1: thrombospondin-1; CTGF: connective tissue growth factor; TIMP: tissue inhibitor of metalloproteinase; ALCAM: activated leukocyte cell adhesion molecule; EHD1: EH-domain containing 1; TLK1: serine/threonine-protein kinase tousled-like 1; Sufu: suppressor of fused; Fus-1: nuclear fusion protein 1; SOCS3: suppressor of cytokine signaling-3; JAK: Janus kinase; STAT: signal transducer of activation; ↑: up-regulation.

**Table 2:** Summary of anti-angiogenic miRNAs involved in tumor angiogenesis regulation.

miRNAs	Targets	Tumor	Function	References
miR-221/222	c-Kit VEGFR-2 ETS-1 PIK3R1 CDKN1B ZEB2 STAT5A	Metastatic renal cell carcinoma, breast cancer	↓ endothelial cell migration, proliferation, and ability to form new capillaries ↓ VEGFR-2	[75,76,77,78,79,80,81,82,83]
miR-126	VEGF-A PIK3R2 ADM	Esophageal cancer, breast cancer, cervical cancer	↓ VEGF/PI3K/AKT signaling ↓ Angiogenesis and microvessel density	[84,85,86]
let-7a	TGFBR3	Pancreatic cancer, hepatocellular carcinoma	↓ tube formation and migration of endothelial cells	[87,88,89,90]
miR-328	CD44	Breast cancer MT1cell line	↓ endothelial cell activity, tubulogenesis and blood vessel formation	[91]
miR-135a	FAK IGF-1/PI3K/Akt pathway	Gastric cancer, non-small cell lung cancer	↓ VEGF signaling	[92,93]
miR-29b	VEGF ERK Akt MMP-2	endometrial carcinoma, breast cancer, pancreatic ductal adenocarcinoma and hepatocellular carcinoma	↓ VEGF expression ↓ MAPK/ERK and PI3K/Akt signaling pathways ↓ MMP-2 expression and VEGFR-2 signaling	[94,95,96,97]
miR-206	VEGF MAPK3 SOX914-3-3ζ/STAT3/HIF-1α/VEGF signaling c-Met/PI3k/Akt/mTOR signaling	laryngeal squamous cell carcinoma, triple negative breast cancer, non-small cell lung cancer	↓ VEGF expression ↓ endothelial cell migration and capillary tube formation	[98,99,100,101]
miR-140-5p	VEGF-A	colorectal cancer, glioma, glioblastoma	↓ VEGF-A/MMP2 signaling	[102,103,104]
miR-497	VEGF-A HIF-1α VEGFR-2	colorectal cancer, breast cancer; renal carcinoma	↓ VEGF-A/ERK/MMP-9 signaling ↓ tube formation and branch points ↓ MEK/ERK and p38 MAPK pathways	[105,106,107]
miR-377	VEGF CD133 Sp1	esophageal squamous cell carcinoma, glioblastoma	↓ VEGF	[108,109]
miR-218	RICTOR Angiopoietin-2 ROBO1	Prostate cancer, gastric cancer	↓ RICTOR/mTOR/HIF-1/VEGF signaling pathway ↓ endothelial cell sprouting, motility and tube formation	[110,111,112]
miR-134	VEGF-A VEGFR-1	osteosarcoma	↓ VEGF-A/VEGFR-1-AKT signaling	[113]

Abbreviations: miRNAs: microRNAs; VEGFR-2: vascular endothelial growth factor receptor2; ETS-1: E26 transformation-specific sequence-1; PIK3R1: phosphoinositide-3-kinase regulatory subunit 1; CDKN1B: cyclin-dependent kinase inhibitor 1b; ZEB2: Zinc finger E-box-binding homeobox 2; STAT5A: signal transducer and activator of transcription 5A; VEGF-A: vascular endothelial growth factor-A; PIK3R2: phosphoinositide-3-kinase regulatory subunit 2; ADM: adrenomedullin; AKT: RAC-Alpha Serine/Threonine-Protein Kinase; PI3K: phosphoinositide 3-kinase; TGFBR3: transforming growth factor beta (TGFβ) receptor III; CD: cluster of differentiation; FAK: focal adhesion kinase; MMP-2: matrix metalloproteinase-2; MAPK3: mitogen-activated protein kinase3; SOX9: Sry-type HMG box 9; Sp1: specific protein 1; ERK: extracellular regulated kinase; mTOR: mammalian target of rapamycin; RICTOR: rapamycin-insensitive companion of mammalian target of rapamycin; ROBO1: roundabout guidance receptor 1; ↓: down-regulation.
